# Basilar artery occlusion presenting as sudden bilateral deafness: a case report

**DOI:** 10.1186/s13256-020-02574-8

**Published:** 2021-03-02

**Authors:** Tomoya Kinouchi, Keisuke Ishitani, Shinichi Uyama, Tadashi Miyamoto, Naomi Fujimoto, Hiromi Ueta

**Affiliations:** 1 Department of Neurosurgery, Tokushima Municipal Hospital, 2-34, Kitajyosanjima-cho, Tokushima, 770-0812 Japan; 2 Department of Otolaryngology, Takamatsu Red Cross Hospital, Kagawa, Japan; 3 Department of Neurosurgery, Tokushima Kensei Hospital, Tokushima, Japan

**Keywords:** Sudden bilateral deafness, Bilateral sensorineural hearing loss, Vertebrobasilar artery occlusion, Endovascular treatment

## Abstract

**Background:**

Most sudden-onset hearing loss is due to otolaryngologic- and very rarely to cerebrovascular disease. We report a woman with sudden bilateral sensorineural hearing loss. This case suggests that even in the absence of brainstem or cerebellar signs, magnetic resonance imaging (MRI) and MR angiography (MRA) should be performed since such studies may reveal signs of life-threatening vertebrobasilar artery occlusion.

**Case presentation:**

A 73-year-old Japanese woman with a history of hypertension, hyperlipidemia, and atrial fibrillation who suffered bilateral deafness with vertigo and vomiting was transferred from a local hospital to our department. On admission her consciousness was clear and vertigo was absent. Neurological examination revealed only bilateral sensorineural hearing loss. Head computed tomography (CT) returned no significant findings. The next morning she gradually developed severe drowsiness. Diffusion-weighted MRI demonstrated acute cerebral infarction in the brainstem and bilateral cerebellum; MRA showed basilar artery occlusion due to a cardioembolic thrombus. Revascularization was obtained by endovascular treatment. However, her condition worsened progressively during the following hours. CT revealed new brainstem lesions, massive cerebellar swelling, and obstructive hydrocephalus. She died on the second day after her admission.

**Conclusions:**

When hearing loss is due to vertebrobasilar occlusive disease, the prognosis is very poor. We suggest that vertebrobasilar stroke be suspected in patients with bilateral sensorineural hearing loss who present with risk factors for stroke such as atrial fibrillation and other neurologic signs.

## Introduction

The prognosis of patients with sudden-onset sensorineural hearing loss, an inner ear disorder, is relatively good. It tends to be due to idiopathic sudden deafness, Meniere's disease, or a perilymphatic fistula. More rarely it is attributable to vertebrobasilar ischemia. The incidence of vertebrobasilar ischemia in sudden sensorineural hearing loss is approximately 1.2% [1]. The anterior-inferior cerebellar artery (AICA) which originates from the basilar artery (BA) is implicated in acute audiovestibular dysfunction. Sudden bilateral deafness due to AICA ischemia is associated with multiple brainstem signs or symptoms and rarely with a single factor.

When hearing loss is due to vertebrobasilar occlusion, the prognosis is very poor. We report a woman who presented with only bilateral hearing loss without vertigo and vomiting. Consequently, initially we did not suspect specific anomalies including vertebrobasilar impairment. However, angiography revealed occlusion of lower third of the BA. Based on the experience reported here, we suggest that patients with sudden bilateral hearing loss be subjected to MRI and MRA studies because they may reveal signs of life-threatening vertebrobasilar artery occlusive disease.

## Case presentation

A 73-year-old Japanese woman with a history of hypertension, hyperlipidemia, and atrial fibrillation (AF) with a CHA2DS2-VASC score of 3 developed bilateral hearing loss with vertigo and vomiting and was brought to a local hospital. Her home medications included 5 mg daily of atorvastatin. The diagnosis was sudden deafness and she was transferred to our emergency department. At that time her consciousness was clear. She had no significant family, social, environmental, or employment history. She did not smoke or take alcohol. Her blood pressure was 147/72 mmHg, her pulse rate was 78 beats/minute with AF, and her temperature 36.8 °C. One year earlier she interrupted anticoagulant therapy because she experienced recurrent epistaxis.

**Fig. 1.**
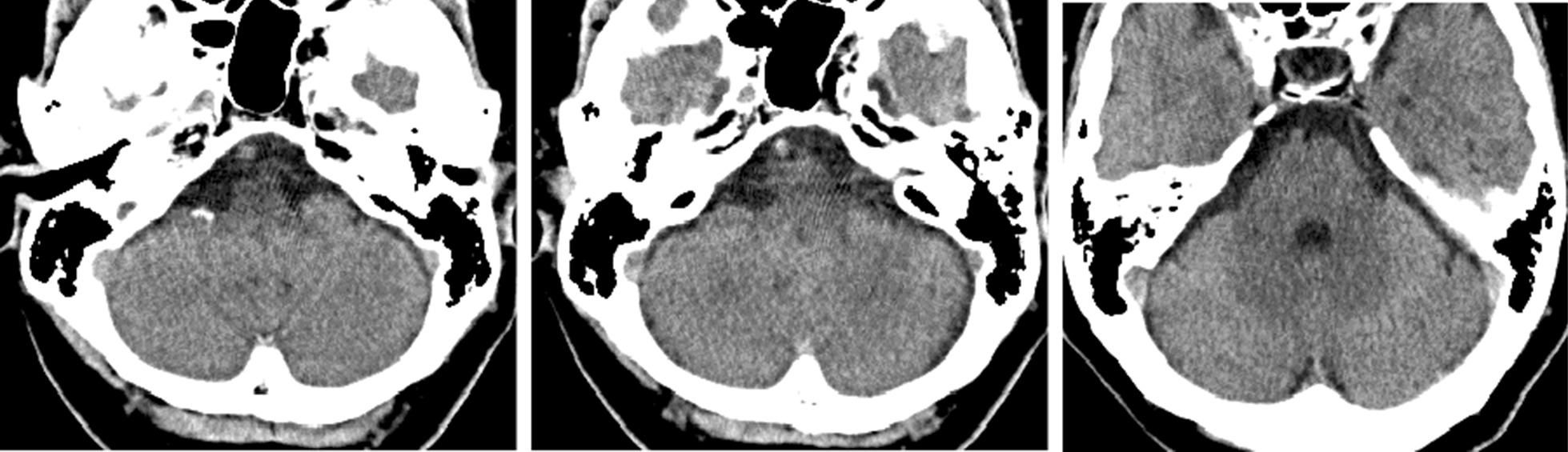
Brain computed tomography showed no new lesions in the area of the cerebellar hemispheres and brainstem

She reported only bilateral hearing loss without vertigo and vomiting. Dysarthria, weakness, ataxia, diplopia, dysphagia, and Horner syndrome were absent. As computed tomography (CT) returned no specific findings (Fig. 1) she returned home and was scheduled for audiometry on the next day.

**Fig. 2.**
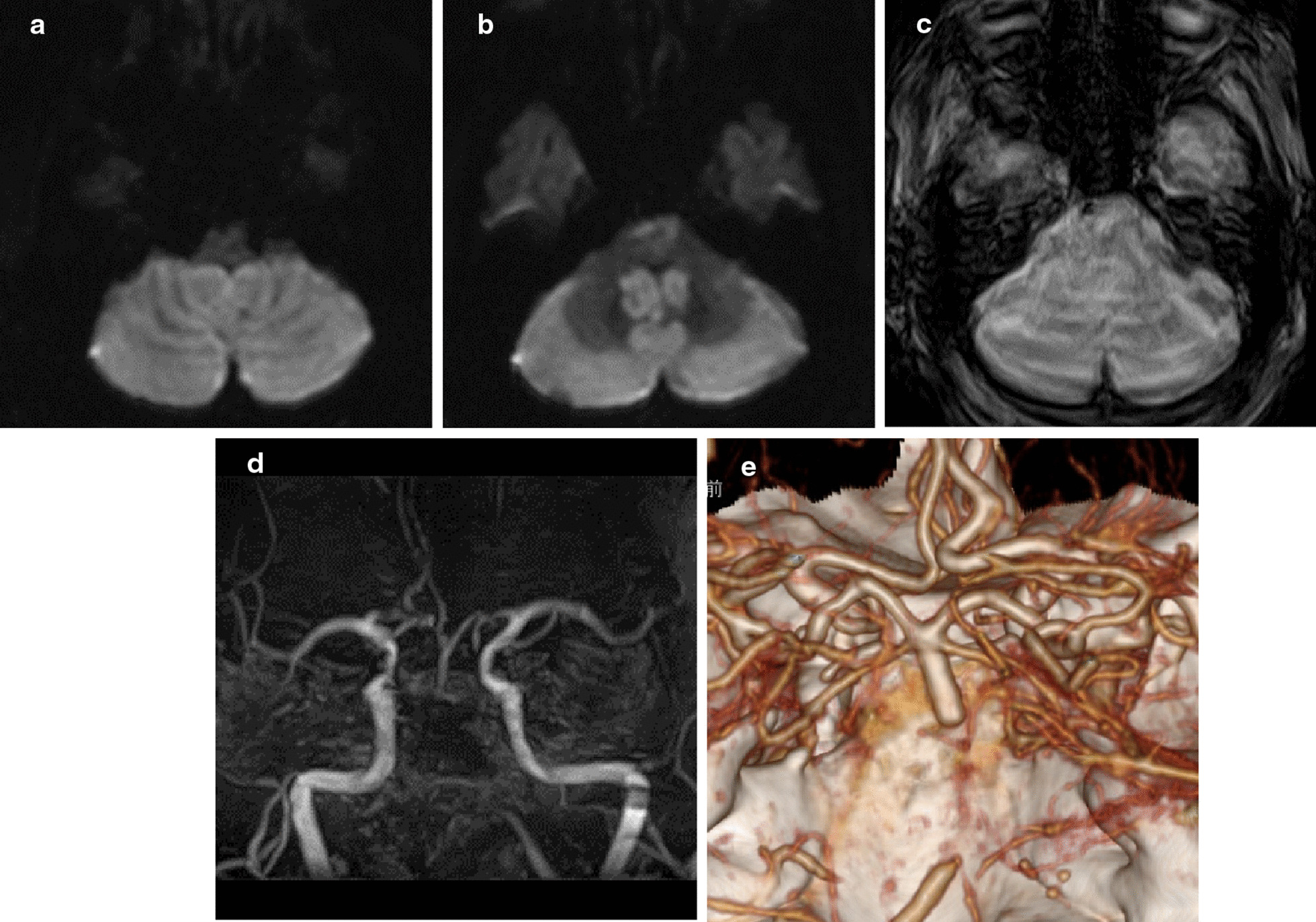
Diffusion-weighted brain MRI showing acute multifocal lesions involving the bilateral cerebellar hemispheres (**a**, **b**). The susceptibility vessel sign was noted in the middle portion of the basilar artery on the T2*-weighted image (**c**). The V3 segment of the right VA, the V4 segment of the left VA, and the lower third of the BA were occluded on MRA and 3D-CTA images (**d**, **e**)

The next morning her consciousness decreased progressively and she returned to our hospital. At the time of admission she was severely drowsy. Her Glasgow Coma Scale (GCS) score was 7 (E2V1M4) and the National Institute of Health Stroke Scale (NIHSS) score was 33. We found the normal pupil reflexes, symmetrical facial responses and withdrawal of both arms and legs to painful stimuli. Her general physical examination was unremarkable. Her blood pressure was 170/90 mmHg, her pulse rate was 80/minute with AF, and her temperature 36.4 °C. Her laboratory test results showed a white blood cell count of 8.8 × 10^9^/l, hemoglobin of 14.7 g/dl, platelet count of 268 × 10^9^/l, blood urea nitrogen 11.3 mg/dl, and creatinine of 0.58 mg/dl, as well as a normal liver function test result. Diffusion-weighted imaging (DWI) revealed acute multifocal lesions involving the bilateral cerebellar hemispheres and pons and the posterior circulation. The DWI posterior circulation Acute Stroke Prognosis Early CT Score (DWI-pc-ASPECTS) was 7 (Fig. 2a, b). The susceptibility vessel sign was noted in the BA on T2*-weighted images (Fig. 2c). Magnetic resonance angiography (MRA) and 3D CT angiography (CTA) showed occlusion of the V3–4 segments of the bilateral vertebral arteries (VA) and the BA (Fig .2d, e). As conservative treatment was thought to be ineffective we started edaravone infusion (30 mg intravenously, twice a day) and performed endovascular treatment.

**Fig. 3.**
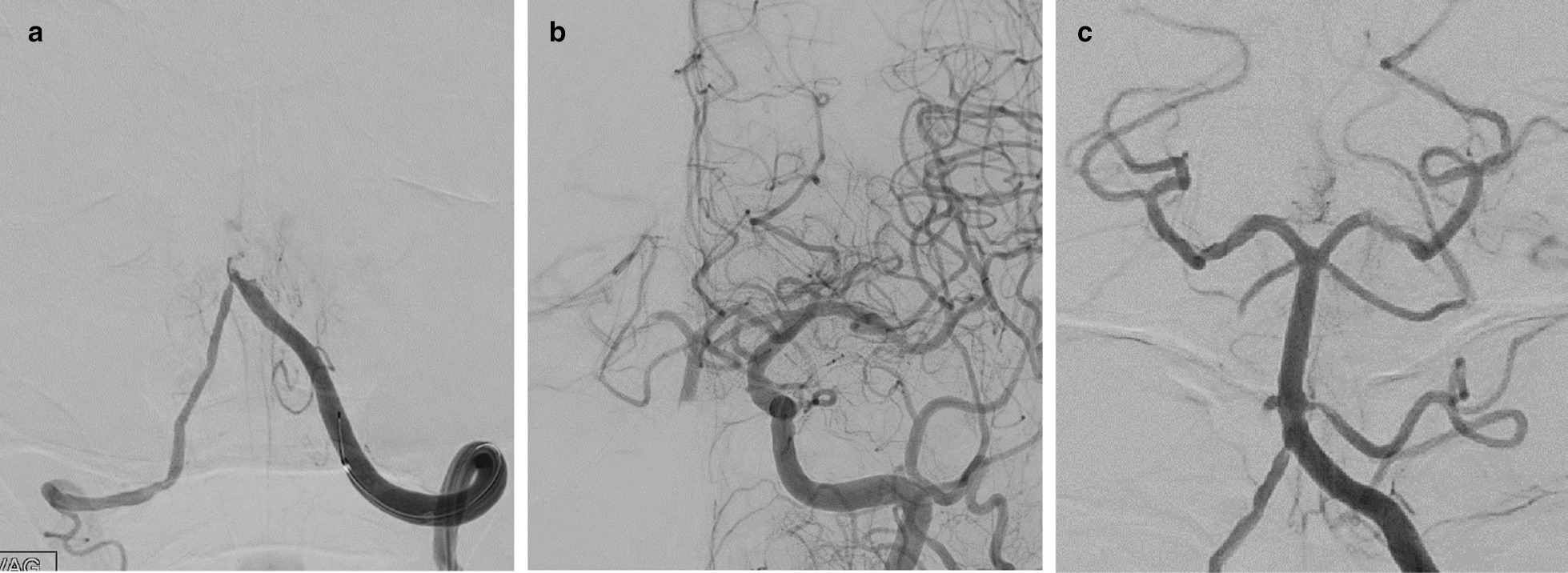
Anteroposterior left vertebral angiography revealed BA trunk occlusion (**a**). Anteroposterior left common carotid angiograms showed reverse flow into the BA and SCA through the PcomA (**b**). Post-thrombectomy, the last anteroposterior left vertebral angiogram demonstrated total recanalization of the BA trunk and residual occlusion of the right AICA and SCA (**c**)

Transfemoral cerebral angiography showed occlusion of the V4 segment of the left VA just proximal to its union with the BA (Fig. 3a, b). Left common carotid angiography demonstrated retrograde blood flow into the BA and the bilateral superior cerebellar artery (SCA) via the left posterior communicating artery (PcomA); the proximal side was obstructed to the union area. Two-pass 5MAX ACE (Penumbra Inc., Alameda, USA) using a direct aspiration first-pass technique was successful. While the BA trunk was completely reperfused, the right AICA and SCA remained occluded (Fig. 3c).

**Fig. 4.**
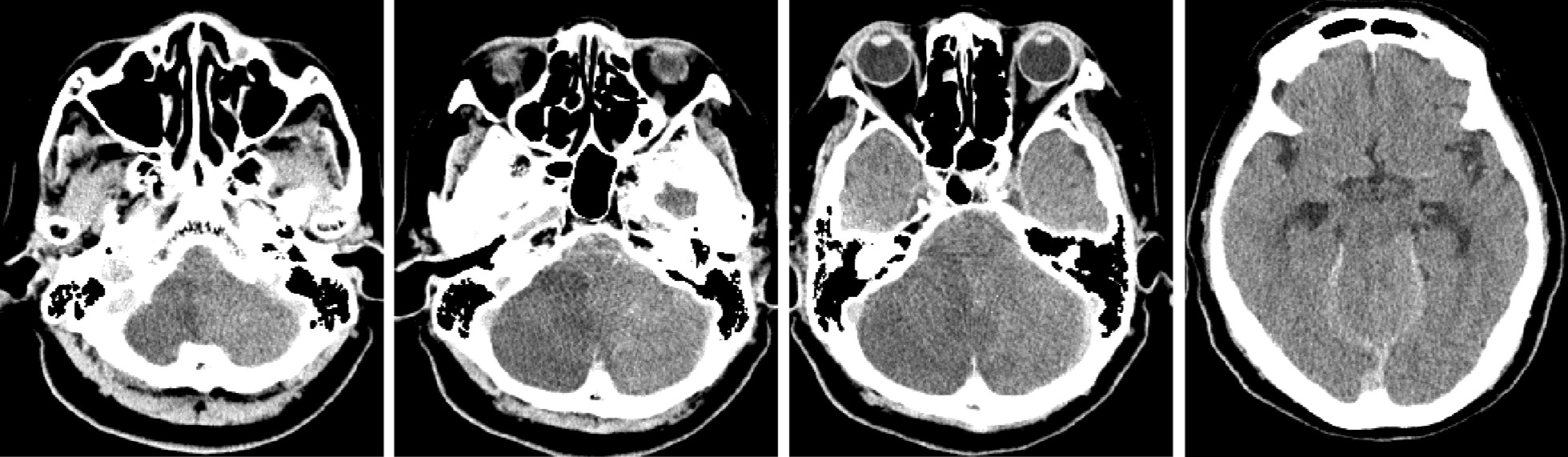
Postoperative brain CT demonstrated progressive infarction and transtentorial upward herniation

Her past history of untreated AF and current angiographic findings strongly suggested a cardioembolic thrombus-induced basilar artery occlusion; we did not perform echocardiography. Postoperatively she was comatose; brain CT showed a large brainstem infarct and upward herniation (Fig. 4) and she died on the second day after admission. Whole body and brain autopsy was not performed.

## Discussion

We showed a woman who presented with only bilateral hearing loss without vertigo and vomiting caused by embolic occlusion of lower third of the BA. When hearing loss is due to vertebrobasilar occlusion, the prognosis is very poor. We suggest that patients with sudden bilateral hearing loss be subjected to MRI and MRA studies because they may reveal signs of life-threatening vertebrobasilar artery occlusive disease.

Sudden-onset sensorineural hearing loss usually suggests an inner ear disorder, e.g. Meniere disease, acute labyrinthitis, autoimmune inner ear disease, or a perilymphatic fistula. Most sudden-onset sensorineural hearing loss is unilateral. The reported incidence of vertebrobasilar ischemia in patients with sudden sensorineural hearing loss is approximately 1.2–8.0% [1, 2]. Vertebrobasilar artery occlusion was implicated in 6% of patients with acute sensorineural hearing loss [3]; 1.4% of patients with vertebrobasilar impairment presented with bilateral hearing loss [4]. Strokes associated with BA occlusion elicit numerous neurological symptoms or signs. Huang *et al.* [4] reported 7 patients with bilateral sudden deafness due to vertebrobasilar occlusive disease, 4 of them suffered vertigo only at onset. We suspect that vertebrobasilar ischemia is often overlooked.

Our patient had no symptoms of brainstem and cerebellar involvement. Among vascular causes, ischemic stroke in the territory of the AICA, a branch of the BA, is the leading cause of sensorineural hearing loss [5, 6]. Usually the internal auditory artery (IAA) originates from the AICA and the inner ear receives its sole supply from the IAA. Strokes in the AICA territory have been shown to be associated with occlusion of a BA branch [7–9]. At the time of admission to our hospital, our patient presented with only bilateral hearing loss without vertigo and vomiting, consequently, initially we did not suspect specific anomalies including vertebrobasilar impairment. However, angiography revealed occlusion of lower third of the BA.

Misery perfusion in the bilateral AICA territories was due to insufficient supply from the PcomA-mediated retrograde collateral pathway. Because the inner ear requires a high-energy metabolism and the IAA is an end artery with little collateral circulation from the otic capsule, the inner ear is particularly vulnerable to ischemia [10–14]. The vestibular structure is relatively well supplied by a rich network of anastomosing vessels from the posterior inferior cerebellar artery (PICA), the SCA, and the VA [15–17]. AICA hypoperfusion associated with BA occlusion can result in selective damage and elicit cochlear dysfunction, leading to bilateral sensorineural hearing loss. Our patient suffered acute progression of ischemia to the bilateral cerebellum and the pons of the posterior circulation and she manifested severe symptoms of brainstem involvement.

Many patients with vertebrobasilar occlusive disease have a poor outcome and experience truncal ataxia, quadriplegia, locked-in syndrome, coma, and death [4, 18, 19]. Before the onset of transient vertigo, nausea and/or tinnitus, 8–16% of patients with AICA territory infarction manifest acute audiovestibular disturbance [4, 11, 20]. Therefore, especially older patients with sudden-onset sensorineural hearing loss and episodic central nervous system symptoms or signs and a history of atherosclerosis or embolism must be carefully evaluated. Our patient had a history of untreated AF. The delivery of recombinant tissue plasminogen activator (rtPA) has been the standard of care in patients with acute ischemic stroke. However, rtPA must be administered within 4.5 h of stroke onset. To address acute ischemic stroke due to intracranial large-vessel occlusion, endovascular treatment by mechanical thrombectomy has been recommended [21, 22]. A meta-analysis [23] found that endovascular thrombectomy was effective when performed within 6–24 h after the onset of ischemic stroke as long as the region was still ischemic and not yet infarcted. There is no sufficient evidence for the effectiveness of this treatment in patients with BA occlusion. We found no recommendations for using both rtPA and endovascular thrombectomy at the posterior circulation in our patient. Yoon *et al.* [24] and Kim *et al.* [25] reported that they obtained a good clinical outcome when they treated patients with a DWI-pc-ASPECTS of 6 or less by endovascular reperfusion therapy. Although endovascular treatment revascularized the vertebrobasilar artery in our patient, it was too late to prevent the expansion of irreversible ischemic damage. We suggest that the early diagnosis and proper management of audiovestibular events may provide a window to prevent the progression of infarction to larger areas of the posterior circulation.

## Conclusion

Ours is a rare case of sudden bilateral sensorineural hearing loss due to embolic occlusion of the vertebrobasilar artery, the AICA, and the PICA. The delayed diagnosis of ischemic stroke in the posterior circulation can be life-threatening. Therefore, an early diagnosis and the proper management of hearing impairment may provide a window to prevent the progression of infarction to larger areas of the brainstem and cerebellum. Clinicians must consider the possibility of vertebrobasilar occlusive disorder especially in patients with sudden bilateral hearing impairment, with risk factors for stroke and the manifestation of other neurologic signs.

## Data Availability

Not applicable.

## References

[1] Sauvaget E, Kici S, Petelle B, Kania R, Chabriat H, Herman P, et al. Vertebrobasilar occlusive disorders presenting as sudden sensorineural hearing loss. Laryngoscope. 2004;114:327–32. 10.1097/00005537-200402000-00028 14755213

[2] Lee H, Baloh RW. Sudden deafness in vertebrobasilar ischemia: clinical features, vascular topographical patterns and long-term outcome. J Neurol Sci. 2005;228:99–104. 10.1016/j.jns.2004.10.016 15607217

[3] Ferbert A, Brückmann H, Drummen R. Clinical features of proven basilar artery occlusion. Stroke. 1990;21:1135–42. 10.1161/01.str.21.8.1135 2389292

[4] Huang MH, Huang CC, Ryu SJ, Chu NS. Sudden bilateral hearing impairment in vertebrobasilar occlusive disease. Stroke. 1993;24:132–7. 10.1161/01.str.24.1.132 8418537

[5] Lee H. Neuro-otological aspects of cerebellar stroke syndrome. J Clin Neurol. 2009;5:65–73. 10.3988/jcn.2009.5.2.65 PMC2706413 19587812

[6] Lee H, Sohn SI, Jung DK, Cho YW, Lim JG, Yi SD, et al. Sudden deafness and anterior inferior cerebellar artery infarction. Stroke. 2002;33:2807–12. 10.1161/01.str.0000038692.17290.24 12468774

[7] Amarenco P, Rosengart A, DeWitt LD, Pessin MS, Caplan LR. Anterior inferior cerebellar artery territory infarcts Mechanisms and clinical features. Arch Neurol. 1993;50:154–61. 10.1001/archneur.1993.00540020032014 8431134

[8] Caplan LR. Intracranial branch atheromatous disease: a neglected, understudied, and underused concept. Neurology. 1989;39:1246–50. 10.1212/wnl.39.9.1246 2671793

[9] Caplan LR, Rosenbaum AE. Role of cerebral angiography in vertebrobasilar occlusive disease. J Neurol Neurosurg Psychiatry. 1975;38:601–12. 10.1136/jnnp.38.6.601 PMC492036 1151428

[10] Grad A, Baloh RW. Vertigo of vascular origin. Clinical and electronystagmographic features in 84 cases. Arch Neurol. 1989;46:281–4. 10.1001/archneur.1989.00520390047014 2919982

[11] Kim HA, Lee H. Recent advances in understanding audiovestibular loss of a vascular cause. J Stroke. 2017;19:61–6. 10.5853/jos.2016.00857 PMC5307938 28030893

[12] Kim JS, Lee H. Inner ear dysfunction due to vertebrobasilar ischemic stroke. Semin Neurol. 2009;29:534–40. 10.1055/s-0029-1241037 19834865

[13] Lee H, Kim JS, Chung EJ, Yi HA, Chung IS, Lee SR, et al. Infarction in the territory of anterior inferior cerebellar artery: Spectrum of audiovestibular loss. Stroke. 2009;40:3745–51. 10.1161/STROKEAHA.109.564682 19797177

[14] Oas JG, Baloh RW. Vertigo and the anterior inferior cerebellar artery syndrome. Neurology. 1992;42:2274–9. 10.1212/wnl.42.12.2274 1461378

[15] Lee H, Cho YW. Auditory disturbance as a prodrome of anterior inferior cerebellar artery infarction. J Neurol Neurosurg Psychiatry. 2003;74:1644–8. 10.1136/jnnp.74.12.1644 PMC1757416 14638883

[16] Mazzoni A. Internal auditory artery supply to the petrous bone. Ann Otol Rhinol Laryngol. 1972;81:13–21. 10.1177/000348947208100103 5009813

[17] Mazzoni A. Internal auditory canal arterial relations at the porus acusticus. Ann Otol Rhinol Laryngol. 1969;78:797–814. 10.1177/000348946907800413 5799404

[18] Kim E, Son MK, Kang CK, Lee YB. Vertebrobasilar occlusion presenting as sudden isolated bilateral sensorineural hearing loss: case report. J Cerebrovasc Endovasc Neurosurg. 2013;15:225–8. 10.7461/jcen.2013.15.3.225 PMC3804662 24167804

[19] Toyoda K, Hirano T, Kumai Y, Fujii K, Kiritoshi S, Ibayashi S. Bilateral deafness as a prodromal symptom of basilar artery occlusion. J Neurol Sci. 2002;193:147–50. 10.1016/s0022-510x(01)00663-3 11790395

[20] Kim JS, Cho KH, Lee H. Isolated labyrinthine infarction as a harbinger of anterior inferior cerebellar artery territory infarction with normal diffusion-weighted brain MRI. J Neurol Sci. 2009;278:82–4. 10.1016/j.jns.2008.12.002 19135217

[21] Berkhemer OA, Fransen PS, Beumer D, MR CLEAN Investigators, et al. A randomized trial of intraarterial treatment for acute ischemic stroke. N Engl J Med. 2015;372:11–20. 10.1056/NEJMoa1411587 25517348

[22] Saver JL, Goyal M, Bonafe A, SWIFT PRIME Investigators, et al. Stent-retriever thrombectomy after intravenous t-PA vs. t-PA alone in stroke. N Engl J Med. 2015;372:2285–95. 10.1056/NEJMoa1415061 25882376

[23] Nogueira RG, Jadhav AP, Haussen DC, DAWN Trial Investigators, et al. Thrombectomy 6 to 24 hours after stroke with a mismatch between deficit and infarct. N Engl J Med. 2018;378:11–21. 10.1056/NEJMoa1706442 29129157

[24] Yoon W, Kim SK, Heo TW, Baek BH, Lee YY, Kang HK. Predictors of good outcome after stent-retriever thrombectomy in acute basilar artery occlusion. Stroke. 2015;46:2972–5. 10.1161/STROKEAHA.115.010840 26330448

[25] Kim JG, Lee D, Choi JC, Song Y, Lee DH, Suh DC. DWI-pc-ASPECT score in basilar artery occlusion: is 6 points or less always indicative of a bad outcome? Interv Neuroradiol. 2019;25:371–9. 10.1177/1591019919827505 PMC6607617 30803336

